# Regional legal and policy instruments for addressing LGBT exclusion in Africa

**DOI:** 10.1080/26410397.2019.1698905

**Published:** 2020-01-13

**Authors:** Chimaraoke Izugbara, Seun Bakare, Meroji Sebany, Boniface Ushie, Frederick Wekesah, Joan Njagi

**Affiliations:** a Director, Global Health, Youth & Development, International Center for Research on Women, Washington, DC, USA . *Correspondence*: cizugbara@icrw.org; b Programs Manager, Amnesty International Nigeria, Abuja, Nigeria; c Gender and Health Specialist, International Center for Research on Women, Washington, DC, USA; d Associate Research Scientist, African Population & Health Research Center, Nairobi, Kenya; e Research Officer, African Population & Health Research Center, Nairobi, Kenya

**Keywords:** Africa, LGBT, African Union, exclusion, regional treaties

## Abstract

The vulnerability of lesbian, gay, bisexual and transgender (LGBT) persons in Africa to public health and other risks is heightened by their exclusion from socio-economic opportunities and services. We analysed existing regional-level legal and policy instruments and treaties for the opportunities they offer to tackle the exclusion of LGBT persons in Africa. We identified seven key living legal and policy instruments, formulated and adopted between 1981 and 2018, by the African Union (AU) or its precursor, the Organization of African Unity. These treaties and instruments do not only highlight the region’s challenges related to inclusion, most of them are binding and enforceable, and all enshrine the responsibility of AU member-states to safeguard and ensure the inclusion and protection of citizens, their gender or sexual orientation notwithstanding. The instruments set forth strong and ambitious agendas of inclusion and recognise and affirm the rights of the region’s citizens to sexual and reproductive health (SRH), equality, freedom and opportunities, regardless of their sexual orientation. Their language is generally universalist and their rejection of discrimination, criminalisation, and denial of socio-economic opportunities and services to the region’s sexual minorities is forthright. However, the instruments do not explicitly mention LGBT persons and lack clear and effective mechanisms for answerability among member-states. Accountability and commitment among member-states towards these instruments and policies will improve national legal and policy environments and propel forward the agenda of LGBT inclusion, SRH and wellbeing in the region.

## Résumé

En Afrique, la vulnérabilité des personnes lesbiennes, homosexuelles, bisexuelles et transgenres (LGBT) aux risques de santé publique et autres est aggravée par leur exclusion des possibilités et services socio-économiques. Nous avons analysé les instruments et traités juridiques et politiques existant au niveau régional pour les occasions qu’ils offrent de s’attaquer à l’exclusion des personnes LGBT en Afrique. Nous avons identifié sept instruments juridiques et politiques majeurs en vigueur, formulés et adoptés entre 1981 et 2018, par l’Union africaine (UA) ou l’Organisation de l’unité africaine qui l’a précédée. Ces traités et instruments ne se contentent pas de mettre en lumière les difficultés relatives à l’inclusion dans la région ; la plupart d’entre eux sont juridiquement contraignants et opposables, et tous consacrent la responsabilité des États membres de l’UA de protéger et garantir l’inclusion et la protection des citoyens, quels que soient leur genre et leur orientation sexuelle. Les instruments exposent des programmes d’inclusion solides et ambitieux ; ils reconnaissent et affirment les droits des citoyens de la région à la santé sexuelle et reproductive, à l’égalité, à la liberté et aux potentialités, quelle que soit leur orientation sexuelle. Leur langue est en général universaliste et ils rejettent nettement la discrimination, la pénalisation et le déni d’accès aux débouchés et services socio-économiques des minorités sexuelles de la région. Néanmoins, ces instruments ne mentionnent pas explicitement les personnes LGBT et des mécanismes clairs et opérants leur font défaut pour demander des comptes aux États membres. La responsabilité des États membres et leur engagement en faveur de ces instruments et politiques amélioreront les environnements juridiques et politiques nationaux, et feront avancer le programme d’inclusion, de santé sexuelle et reproductive et de bien-être des personnes LGBT dans la région.

## Resumen

La vulnerabilidad de personas lesbianas, gay, bisexuales y transgénero (LGBT) en África a riesgos de salud pública y de otra índole es intensificada por su exclusión de oportunidades y servicios socioeconómicos. Analizamos instrumentos legislativos y normativos y tratados a nivel regional por las oportunidades que ofrecen para abordar la exclusión de personas LGBT en África. Identificamos siete instrumentos legislativos y normativos vigentes, formulados y adoptados entre 1981 y 2018, por la Unión Africana (UA) o su precursor, la Organización de Unidad Africana. Estos tratados e instrumentos no solo destacan los retos de la región relacionados con la inclusión; la mayoría de ellos son vinculantes y aplicables, y todos consagran la responsabilidad de los Estados miembros de la UA de salvaguardar y garantizar la inclusión y protección de su ciudadanía, sin importar su género u orientación sexual. Los instrumentos establecen agendas importantes y ambiciosas de inclusión y reconocen y afirman los derechos de la ciudadanía de la región a salud sexual y reproductiva (SSR), igualdad, libertad y oportunidades, independientemente de su orientación sexual. Su lenguaje generalmente es universalista, y su rechazo de discriminación, penalización y negación de oportunidades y servicios socioeconómicos a las minorías sexuales de la región es explícito. Sin embargo, los instrumentos no mencionan explícitamente a las personas LGBT y carecen de mecanismos claros y eficaces para responsabilizar a los Estados miembros. La rendición de cuentas y el compromiso de los Estados miembros con relación a la aplicación de estos instrumentos y políticas mejorarán los entornos legislativos y políticos nacionales e impulsarán la agenda de inclusión de LGBT, SSR y bienestar en la región.

## Introduction

Issues related to the rights of lesbian, gay, bisexual, and transgender (LGBT) persons present unique socioeconomic, public health, and development challenges for many African states.^1^ Pervasive poor understanding of the gender and sexuality-related circumstances of LGBT persons continues to expose them to multiple dangers, poor health outcomes, elevated levels of exclusion from critical social, economic and political processes, and violations that not only often go largely unchallenged and unprosecuted, but have major sexual and reproductive health (SRH) implications.^2^ Homophobia, or protective homophobia,^3^ rooted in lay association of homosexuality with AIDS, pedophilia, immorality and irreligiosity, also remains pervasive in the region, constraining LGBT persons’ access to critical public goods, including essential SRH services. Scholars have argued that being LGBT*, per se,* is not a risk in Africa. Rather, the vulnerability of LGBT persons in the region is firmly rooted in their pervasive social exclusion from, and the resulting inequities in access to, opportunities and services.^4,5^ Tucker et al^6^ showed that depression due to homophobic stigma is a significant concern for township men who have sex with men (MSM) in South Africa, with 56.6% reaching the threshold for at least mild depression. This is the context against which calls have been made for more systematic explorations and contemplations of opportunities for fostering the inclusion of LGBT persons in the region’s socio-economic processes.^7,8^

This paper presents and discusses regional legislative and policy instruments developed and adopted by the African Union that have value for efforts to promote LGBT inclusion in socio-economic development in Africa. We also explore some current applications of these instruments in court rulings and related interventions on LGBT issues in the continent.

Recognising and strengthening opportunities to address exclusion related to sexual orientation and gender identity (SOGI) is key for development, SRH, prosperity, and wellbeing in Africa.^9^ Such knowledge can furnish critical actions and entry points for grasping where the greatest prospects for transformation rest, for underscoring areas where change is needed, and for forging alliances and partnerships to foster change at scale. Opportunities may take a variety of forms, including extant and new policies, discourses, or even data for supporting LGBT persons or providing them with potential options to live better; technological developments that could benefit advocacy and networking; or new grassroot and global movements for boosting public engagement and communication.^10^ Formal legislations could also offer policy and programmatic opportunities to more clearly focus the multiple needs and concerns of LGBT persons, including their elevated risks of being denied access to health care services, particularly SRH services.^11^ This paper commences the task of addressing gaps in evidence on formal opportunities for tackling the exclusion and marginalisation of LGBT persons in Africa. It specifically focuses on regional policy and legal instruments formulated and adopted by the African Union (AU) or its precursor, the Organisation of African Unity, between 1981 and 2018.

The world has barely a decade to realise the grand promises enshrined in the Sustainable Development Goals (SDGs). With the seemingly insurmountable challenges related to attaining the SDGs, including insufficient evidence for development planning, financial and resource constraints, and weak governance and accountability structures, the global community urgently needs more effective strategies to connect the potential of existing opportunities to action, in ways that guarantee support for the most marginalised groups, to hold political leaders accountable, and ensure that no one, indeed, is left behind.^12^

## Why focus on regional legal and policy instrument and frameworks?

The ILO^13^ contends that appropriate legal and institutional frameworks are critical to the realisation of rights to social security and improved livelihoods. Good regional policy and legal instruments or frameworks enshrine privileges in transparent ways and allow persons to stake claims and obtain redress for violations of their rights, including sexual and reproductive rights. They protect people from arbitrary or discretionary decision-making, facilitate access to social protection, and help guarantee equality of treatment. Legal and institutional frameworks articulate the roles and responsibilities for actors who design, deliver, monitor, and enforce protection. They provide a basis for advocacy organisations to frame and organise their work and activities, enhance access to justice and health, promote human rights and fundamental freedoms for all, and support the implementation of strategies to promote human rights. A 2015 statement, jointly issued by several UN bodies, observes that the enshrinement of rights and privileges in legislative documents increases the guarantees of social protection and SRHR for the most disadvantaged and vulnerable groups. It also ensures that rights are guarded from political manipulation and that they receive the commitment of state authorities beyond the lives of individual governments.^14^

International treaties and standards affirm access to justice as both a basic human right and a means to protect other universally recognised human rights.^15^ They increase citizens’ understanding of their rights, enshrining them in collective policies and promoting their delivery and access to citizens. Gloppen^16^ and the Council on Foreign Relations^17^ suggest that, too often, without these rights enshrined in regional and collective legal and policy legislations and frameworks, national enforcement and advocacy will remain weak. The lack of effective human rights protection instruments and frameworks renders marginalised groups vulnerable to abuses and violations. International guidelines and instruments provide a basis for advocacy to empower individuals and communities to assert their rights vis-à-vis the state, help nurture fairer, more accountable justice systems, and strengthen the frameworks that support human rights at national, regional, and international levels. They inspire and trigger efforts to expand access to courts and legal representation as well as the ability to engage effectively with law enforcement officials and make use of informal, non-state justice mechanisms, where required. Civil society also relies on international legal instruments to galvanise support for individuals and communities and offer an effective counterbalance to the powers of the state, especially where countries lack effective national legal systems that can guarantee full access to justice for all citizens.^15^

## LGBT exclusion in Africa

There is growing evidence that LGBT persons all over the world continue to experience violations of their human rights, particularly sexual and reproductive health and rights (SRHR).^11,18–20^ Despite legal and social advances in the past two decades, exclusion and discrimination against persons based on their SOGI remain common. In a four-country study of exclusion from SRH services and violence and rights abuses against MSM in Southern Africa, Zahn et al^21^ showed that 46.7% of men in all the sites reported experiencing at least one human rights abuse. Further, 5.1% had been denied healthcare, 11.6% had been raped, 10.5% had been beaten by the police, and 18.7% had been blackmailed because of their sexual orientation. Additionally, 19.2% were afraid to seek SRH services. Several African countries currently have laws that impact negatively on LGBT communities.^11,22,23^ For instance, homosexual activity among men attracts the death penalty in Sudan, Mauritania, Somali, and parts of northern Nigeria; life imprisonment in Uganda, Tanzania, and Sierra Leone; and long jail terms in Kenya, Malawi, Senegal, and Gambia. In Nigeria, heterosexual family members, allies, and friends who support or aid gay and lesbian men and women risk a 10-year jail sentence. Liberal attitudes and robust constitutional guarantees for sexual minority rights in South Africa and Cape Verde have also failed to fully shield their LGBT communities from discrimination, stigma and violence.^20^

LGBT exclusion promotes poor SRH, poverty and economic disadvantage by stalling their access to services, care and security, social participation, equality, and wellbeing. It denies them voice, recognition, engagement, and, at a more fundamental level, compromises their dignity and self-worth as individuals. The forms and implications of SOGI-related exclusion and mistreatment are far-reaching, extending beyond people’s SRH, to encompass their overall wellbeing, the welfare of their households and communities, and even the economic and social fabric of societies.^18,20,21,24^ A 2014 report stated that “LGBT people throughout Africa face the daily threat of harassment, discrimination, prosecution, denial of care, and violence, and others remain vulnerable to increasingly dangerous and concerted efforts to stoke state-sponsored homophobia and transphobia”.^25^ Currently, only South Africa grants full marriage equality and constitutional protection against discrimination to its LGBT citizens. Other reports show that over 30 African nations currently criminalise same-sex relationships.

While attitudes towards homosexuals and homosexuality vary across Africa, social stigma towards LGBT persons remains prevalent in many countries in the region. Kerrigan^26^ found that “exclusion or ostracism by the family is among the worst fears of LGBT persons in Africa”. For LGBT persons who experience violence and mistreatment, injuries, visits to health personnel, disabilities and deaths are common.^27^ Violence against LGBT groups and persons also erodes their confidence and mental health, hindering their productivity and participation in development activities.^28,29^ SOGI-related exclusion and abuses have significant SRH implications and economic costs. They lower productivity, incomes, and rates of accumulation of human and social capital.^24^ Abused and violated LGBT persons experience emotional distress, and tend to frequently consider, attempt, or carry out suicide.^30,31^ They also suffer post-traumatic stress syndrome, depression, anxiety, and low self-esteem, negative behavioural and health outcomes such as alcohol and drug abuse, heightened SRH morbidity and mortality, sexual risk-taking, and a higher risk of subsequent victimisation.^32^

LGBT victimisation affects families and communities, saps household resources, strains family ties, and depresses family members.^30,33^ To avoid violence, abuse, and stigma, LGBT persons may censor their behaviours to what is acceptable to their aggressors and victimisers, often making them “their own jailers”. Other documented effects of the neglect and mistreatment of LGBT persons in sub-Saharan Africa include: poor health outcomes; forced displacement; low educational attainment; high unemployment rates; poor access to quality livelihoods, housing, health and financial services; exposure to violence including arrests, detention, beatings, ill-treatment, and sexual assault.^18,20,23^ For instance, criminalisation of homosexuality increases HIV service providers’ reluctance to engage with MSM. Clark^18^ writes that health service provision to MSM in Senegal has yet to fully recover from the effects of the 2008 arrest of nine HIV health workers who were then serving the population. In Cameroon, where an estimated 30–50% of MSM are infected with HIV, stigma and discrimination remain the foremost barriers to supportive SRH services to the population. Malawian health providers also refuse to offer SRH services to MSM because they would be seen as aiding and abetting homosexuality.^18^

This paper identifies and highlights key living regional legislative and policy instruments that have value for efforts to promote LGBT inclusion in socio-economic development in Africa. We focus on regional policy and legislative documents and instruments developed and published between 1981 and 2018 by the African Union (AU) or its precursor, the Organisation of African Unity. The identification of these regional instruments was facilitated through online and material library searches, interviews with African legal scholars, and discussions with AU officials and LGBT activists and scholars in Africa. By highlighting these legislations and legal policies, we underscore the grounds they inhere for promoting change, advocacy and engagement; clarify gaps in their constitution and implementation; and point out practical actions for realising their visions.

## Methodology

This paper is primarily a review of existing documents. To identify the policy instruments reviewed in this paper, we searched the websites and online databases of the AU and its organs (such as The African Court on Human and Peoples’ Rights) for publicly available information on key policy documents and legal instruments developed by the continental body since 1981. In addition, we conducted searches of databases such as Equaldex, the University of Southern California LGBTQ Studies Databases, EBSCO information Services, LGBT and Gender Studies Database, and Google Scholar for published research and analyses related to AU policy instruments and rulings related to LGBT rights issues by the African Court on Human and Peoples’ Rights (the African Court) and national courts in the Africa. To search these scholarly databases, we combined terms such as “African Union”, “policy instruments”, “human right treaties”, “judgements/rulings”, and “LGBT in Africa”. Electronic consultations were also held with five African legal scholars, three AU officials, and five LGBT activists and scholars in Africa for information on AU policy instruments and human right treaties that have relevance for LGBT inclusion and wellbeing in the continent. They were also asked for information on identifiable judicial rulings of the African Court and national judiciaries in the region that have referenced these instruments and treaties. Consultations with these key informants were merely for the purpose of identifying relevant documents and instruments and judicial rulings. Their assessments of the judicial rulings and why and how the instruments or treaties offer opportunities for LGBT inclusion were not sought. These key informants were purposively identified and contacted through the networks of one the authors who is a human rights lawyer and legal scholar in the region. This paper is not intended to be a systematic review. The AU policy and legal instruments, and African Court and national judicial rulings included in this paper, were identified through suggestions and guidance by the expert informants that we consulted, available online information, and the authors’ professional knowledge of the fields of law and LGBT issues in Africa.

## Findings

As shown in Table 1, we identified and considered seven relevant and key treaties and policy instruments developed and ratified by the AU or its forerunner, the OAU, between 1981 and 2018. These policy documents include both enforceable treaties (that is, binding legal agreements which African states have collective obligation to implement and be held accountable to) and policy instruments (influential regional governing tools aimed to achieve social, political, economic, health and other targets or objectives). The aspiration to promote inclusion and advance the lives, livelihoods and equality of all citizens, their gender and sexuality notwithstanding, is a common feature of these regional treaties and policy instruments. While LGBT persons are not specifically mentioned in any of these instruments, they (the instruments), nevertheless, still emphasise the everyday concerns of LGBT persons and set forth strong and ambitious visions for inclusivity and practical positive action on the marginalisation and concerns of LGBT persons and communities in Africa.

**Table 1.** Key AU legal and policy instruments with potential for addressing LGBT exclusion in member-states: 1981–2018

Nature of instrument	History	Focus	Key values	Limitations in relations to LGBT
The African Charter on Human and Peoples’ Rights				
Enforceable treaty	Approved June 1981; came into effect October 1986; ratified by every AU member-state.	Human rights and basic freedoms; civil and political rights; economic, social and cultural rights; peoples’ rights and group rights; duties of citizens	Right to self-determination, development, education, health, equality of all persons before the law, freedom from discrimination, life and personal integrity, freedom from cruel, inhuman or degrading treatment or punishment, rights to due process concerning arrest and detention, freedom of association, freedom to assembly etc.	No specific mention of vulnerable groups, including LGBT, weak or non-existent monitoring mechanism
African Charter on Democracy, Elections and Governance.				
Enforceable treaty	Adopted January 2007	Democracy and people’s participation as individual fundamental rights	Human rights, rule of law democratic principles, good governance, elimination of forms of discrimination, promoting freedom of expression, citizens’ full participation to development processes, protecting social groups with special needs, improving access to basic social services, ensuring education and literacy.	No specific mention of vulnerable groups, including LGBT; weak monitoring of national implementation relative to the Charter, not ratified by all member-states
African Youth Charter				
Enforceable treaty	Endorsed and adopted July 2006, entered into force August 2009	Strategic youth participation, empowerment and development activities across Africa.	Freedom of movement, expression, private life and property, right to employment, right to education of good quality and that the multiple forms of education, right to equitable and ready access to medical assistance and health care, information, communication and awareness creation, elimination all forms of discrimination against girls and young women	No specific mention of vulnerable groups, including LGBT; weak monitoring of national implementation relative to the Charter, Not ratified by all member-states
The Maputo Protocol (also known as The Protocol to the African Charter on Human and Peoples’ Rights on the Rights of Women in Africa)				
Enforceable treaty	Adopted in July 2003, Came into effect in Nov. 2005	Women’s of civil and political, economic, health, sexual, reproductive, social, cultural environmental rights, reduction of discrimination and infringement of human rights	Equality, freedom, dignity, elimination of gender-based abuse and discrimination	Does not deal directly with discrimination on the basis of sexual orientation or gender identity, no clear definition of sexual rights
The Maputo Plan of Action 2016–2030 for the Operationalisation of the Continental Policy Framework For Sexual and Reproductive Health and Rights				
Enforceable treaty	Launched in 2015 following the expiration of the Maputo Plan of Action for The Operationalisation of the Continental Policy Framework for Sexual and Reproductive Health and Rights 2007-2010, later extended to 2015	Women’s SRHR, empowerment, individual dignity, and welfare and the right to health.	SRHR of men, women, boys and girls and vulnerable and marginalised groups/populations	No clear definition of sexual rights, emphasis on age-appropriate and culturally sensitive comprehensive education on SRH for young people that involves parents and communities, no focus on comprehensive sexuality education, no clear meaning of marginalised groups
Common African Position on the Post-2015 Development Agenda				
AU declarative/obligatory policy instrument	African Union sponsored document published in March 2014	Structural economic transformation inclusive growth, Science& technology People-centred development, Environmental sustainability, Natural Resource Management and Disaster Risk Management, Peace and Security, and Finance and Partnerships.	Inclusivity, reduction in inequality, eradication of poverty, Gender equality and women’s empowerment, universal and equitable access to quality healthcare	Does not deal directly with discrimination on the basis of SOGI no clear definition of sexual rights
AGENDA 2063; The Africa We Want.				
AU declarative/obligatory policy instrument	AU policy roadmap signed May 2013 by African heads of state and government	Gender equality, elimination of GBV discrimination, barriers to quality health and education Ending systemic inequalities, young people Elimination of youth unemployment	Gender parity in public and private institutions, universal access to social, health and economic rights	No specific mention of LGBT persons; Does not deal with or mention discrimination on the basis of sexual orientation or gender identity

Below, we briefly discuss some of the instruments identified in Table 1, highlighting why and how they hold out promise in the region’s quest for LGBT-inclusive development. Given space constraints, we will focus only on four of them: The African Charter, The Maputo Protocol, The African Youth Charter, and The Common African Position (CAP). As we show below, underlying these treaties, policies and instruments are the core values of equality, non-discrimination, and respect for human dignity for all persons and citizens of the region.

### The African Charter on Human and Peoples’ Rights

The African Charter, adopted in 1981 and ratified by all African countries except Sudan, is one of the region’s most influential legal treaties. It grants rights to everyone without distinction. In 12 of its provisions, the Charter asserts the rights of “every individual”. It consistently uses terms such as “every human being”, “no one” and “every citizen” to affirm its inclusive scope of rights holders and assert the notion that individuals should not cease to enjoy rights on grounds of their sexual orientation or gender identity.^34^ Article 2 of the Charter stipulates that “[E]very individual shall be entitled to the rights and freedoms recognized and guaranteed in the Charter without distinction of any kind”. It unambiguously excludes “race, ethnic group, color, sex, language, religion, political or any other opinion, national and social origin, fortune, birth or other status” as reasons for discriminating against any citizens of the region. In Articles 3(1) and (2), the Charter notes that “[E]very individual shall be equal before the law [and] … shall be entitled to equal protection of the law”. This is further clarified in Article 4, which asserts the inviolability of the human being noting that “Every human being shall be entitled to respect for his life and the integrity of his person. No one may be arbitrarily deprived of this right”. Going by the universalist language of the Charter, “[P]rohibited grounds of discrimination include but are not limited to race, ethnic group, colour, sex, gender, sexual orientation, language, religion, political or any other opinion, national and social origin, economic status, birth, disability, age or other status”.^34^ Murray and Viljoen^35^ note that the right to [non-discrimination based on] sexual orientation and gender identity are inherent in the African Charter and that it grants rights to all, regardless of sexual orientation and gender identity.

## Recent applications of the African Charter

The Charter is one of the most applied international treaties in Africa. It has informed landmark resolutions and rulings that have direct implications for the marginalisation of LGBT persons in relation to SRH services and socio-economic opportunities in the region. In its historic resolution adopted during its 55th Ordinary Session (“Resolution 275”), the African Commission offered its strongest condemnation of acts of violence, discrimination and other human rights violations against persons based on SOGI, asserting such acts as violating state obligations under the Charter. Ibrahim^36^ opines that Resolution 275 is the stoutest demonstration of the African Commission’s acknowledgement of the freedoms and rights of LGBTs to full citizenship. In *Attorney General of Botswana v. Thuto Rammoge and 19 others*, the Court of Appeal of Botswana in 2016 ruled that:

“Members of the gay, lesbian and transgender community, although no doubt a small minority, and unacceptable to some on religious or other grounds, form part of the rich diversity of any nation and are fully entitled in Botswana, as in any other progressive state, to the constitutional protection of their dignity.”^37^

The Court upheld the argument that the right to freely associate with others is protected by Article 10 of the African Charter on Human and Peoples Rights and other international instruments. It affirmed that sexual orientation offers no grounds for denying citizens their rights to dignity, freedom, and access to resources and services in Botswana. The High Court in Botswana also recently struck down the provisions of sections 164, 165 and 167 of the country’s penal code which criminalise gay sex. In arriving at the ruling, the court, *inter alia*, referred to the provisions of the African Charter.^38^ In Kenya, where intimidation and violence against LGBT persons remain common, the High Court declared in 2015 that the words “Every person” in Article 36 of the [Kenyan] Constitution include all persons living within the Republic of Kenya despite their sexual orientation. The court quoted Article 10 of the African Charter and reasoned that “[T]hese provisions clearly include all individual, natural persons, and there is nothing to indicate that sexual orientation is a matter that removes one from the ambit of protection by the Constitution”.^39^ Also, in *COI and anor v. Chief Magistrate Ukunda Law Courts and 4 others*,^40^ the Appeal Court in Kenya ruled in 2016 that forced anal examinations for homosexuals are illegal, quoting Article 5, paragraph 24, of the Charter that, regardless of other status, every human being deserves respect for their dignity.^41^ See also, Eric Gitari v NGO Board & 4 others.^42^

The African Commission on Human and People’s Rights and its subsidiary mechanisms have also continued to interpret and apply the provisions of the African Charter in ways that showcase the universalist language and intentions of the Charter. These interpretations continue to specifically clarify the African Charter’s intention to leave no one behind, avert the marginalisation and exclusion of LGBT persons, and ensure the rights and privileges of persons and groups at risk of discrimination and mistreatment based on sexual orientation. Consider for instance that in 2006, and with specific reference to the case *of Zimbabwe Human Rights NGO Forum v. Zimbabwe*, the African Commission affirmed that the “aim of this principle is to ensure equality of treatment for individuals irrespective of nationality, sex, racial or ethnic origin, political opinion, religion or belief, disability, age or sexual orientation”.^43^ For instance, following its 2016 review of the human rights record of Namibia, the African Commission called on the country’s leadership to end the discrimination and stigmatisation that limits health care access for vulnerable groups, particularly the LGBT community, in line with the provisions of the African Charter.^43^ Also, in 2017, the African Commission issued General Comment No. 4 on torture and inhumane and degrading treatment, which explicitly acknowledged the need for acts of sexual and gender-based violence against “lesbian, gay, bisexual, transgender, and intersex persons” to be addressed by African State Parties.^44^ The Commission’s statement reads:

“Any person regardless of their gender may be a victim of sexual and gender-based violence. There is wide prevalence of sexual and gender-based violence perpetrated against women and girls. Acts of sexual violence against men and boys, persons with psychosocial disabilities, and lesbian, gay, bisexual, transgender and intersex persons are of equal concern, and must also be adequately and effectively addressed by State Parties.” (*African Commission on Human and Peoples’ Rights General Comment no. 4 page 18, paragraph 59*)

In the same year (2017), the Commission issued the “Guidelines for the Policing of Assemblies by Law Enforcement in Africa”, which explicitly refers to protections based on sexual orientation and gender identity.^45^ As part of the subsidiary mechanisms of the African Commission, another 2017 report by the African Commission’s Expert Committee on the rights of PLHIV entitled “HIV, the Law and Human Rights in the African Human Rights System: Key Challenges and Opportunities for Rights-Based Responses” explicitly identified the LGBT community as a key population in need of “specific protection and access to HIV and health services”. The report recommended law reform and harm reduction to address age-long abuses of the rights of the group.^46^ Still in 2017, the Special Rapporteur on Human Rights Defenders in Africa also presented a report to the African Commission highlighting the harassment experienced by human rights defenders working on issues related to sexual orientation and gender identity and calling for the repeal of laws undermining freedom of association and discriminating against those fighting for queer rights.^47^ Similarly, the 2017 “Draft Principles on the Declassification and Decriminalization of Petty Offences in Africa” presented by the Special Rapporteur on Prisons, Conditions of Detention and Policing in Africa called for the removal of laws criminalising same-sex sexual conduct.^48^ The 2017 activity report of the Committee for the Prevention of Torture in Africa to the African Commission also called for states to protect those at heightened risk for torture, specifically referencing and including LGBTI persons.^49^

## The Maputo Protocol

The Maputo Protocol is another very critical and enforceable AU policy and legal instrument. Adopted at the 2nd Ordinary Session of the Assembly of the AU in July 2003, the Protocol’s publicly enunciated goal is to “ensure that the rights of women are promoted, realized and protected … to enable them to enjoy fully all their human rights”. The Maputo Protocol guarantees non-discrimination, equitable gender inclusion, and respect for human dignity for all women in Africa. Through its Articles 2, 3 and 4, the Protocol provides for the elimination of all forms of discrimination against women irrespective of their sexual orientation, and requests states to “support the local, national, regional and continental initiatives directed at eradicating all forms of discrimination against women”; guarantees right to dignity; and asserts the rights to life, integrity and security of all women and girls. In the Revised Maputo Plan of Action, published in 2015, the AU offered added impetus and vision for the inclusion of marginalised groups in Africa, including LGBT persons. The influential potential of the Maputo Protocol in striving for LGBT inclusion in Africa is clearly enunciated in one of the over-arching goals of the Revised Plan to “contribute to the attainment of the sexual and reproductive health and rights (SRHR) targets set out in the SDGs”. The Revised Plan calls for investments in vulnerable and marginalised populations and in efforts to ensure that no groups or vulnerable populations are left behind. Paragraph 17(iv) of the Revised Plan succinctly mandates States to:

“[p]rotect the rights of women, men, adolescents and youth to have control over and decide freely and responsibly on matters related to SRH, free from discrimination and violence; eradicate and eliminate all forms of discrimination and violence … ; and promote social values of equality, non-discrimination.”

As an enforceable regional treaty, the Maputo Protocol and its Revised Plan offer yet another strong ground for demands and actions for LGBT inclusion in Africa.

## The African Youth Charter

The African Youth Charter endorsed by the AU in July 2006 is also a key regional enforceable legal instrument. The Charter firmly guarantees enjoyment of rights to all youths, irrespective of colour, sex … and “other status”. The “other status” mentioned in the Youth Charter includes sexual and gender orientations, suggesting that the African member-state signatories of the Charter have no basis or grounds to discriminate against LGBT youth or criminalise their activities and lifestyles as LGBT persons. The Youth Charter has far-reaching and profound significance for LGBT youth in Africa who are particularly at risk for exclusion, harm, and denial of SRH services.^50^ The Youth Charter offers one of the clearest regional-level commitments to the rights, health and wellbeing of youth in Africa. Article 2(2) of the Charter clearly commits member-states to ensure that no youth suffers discrimination based on their status, activities, opinions or beliefs. In its Article 11, the Charter guarantees all young people, irrespective of their SOGI, the right to full participation in all spheres of society. By affirming the social, economic, health and other rights of the region’s youth “irrespective of color, sex … and ‘other status’”, the Youth Charter offers critical grounds for safeguarding the wellbeing and participation of young people in Africa.

## The Common African Position (CAP)

Launched in March 2014, the Common African Position (CAP) on Post-2015 Development Agenda is a critical regional policy instrument. The CAP matters immensely in the drive towards LGBT inclusion in Africa because it unequivocally reiterates the importance of prioritising structural transformation for inclusive and people-centred development in Africa. In several of its provisions, the CAP calls for the inclusion and equality of all persons. Consider paragraph 66 of the CAP which commits member-states to “fight against all forms of discrimination”; paragraph 93 which focuses on diversity and the urgent need to fight discrimination, and paragraph 21, which obligates governments in the region to drive inclusive growth that reduces inequality for all. The CAP offers immense potential to facilitate actions and efforts to ensure that young LGBT citizens of the region are not left behind in development planning, efforts and processes.

The AU’s Agenda 2063 and the African Charter on Democracy, Elections and Governance are other critical regional legal and policy instruments that hold out immense potentials for addressing LGBT exclusion in Africa through their universalist language, inclusive focus, and non-discrimination stance, and firm support for the protection of marginalised groups and peoples, particularly LGBT peoples.

## Discussion

Across much of Africa, LGBT persons are effectively excluded from socio-economic and other opportunities in society. Exclusion of LGBT persons exacerbates the concerns and challenges related to SRH, wellbeing, and development in Africa. It causes and sustains stigma, discrimination, marginalisation, poverty, poor health, and lack of opportunities among LGBT persons. Ensuring the safety and SRH of LGBT persons, as well as their freedom of expression, association, and assembly will help achieve sustainable development in Africa. Current and future development in the region would be compromised by the exclusion of many of its citizens from the development process. The imperative for understanding existing opportunities for promoting the inclusion of the region’s LGBT persons in development is therefore urgent. This paper explored critical opportunities for LGBT inclusion in Africa focusing on existing regional legal and policy instruments that offer a platform for ensuring that LGBT persons in the region are not left behind of development strides and processes.

Seven key regional and policy instruments formulated and endorsed by the AU or OAU between 1981 and 2017 were identified in this review. These instruments, in different ways, recognise and affirm the rights of all the region’s citizens to equality, freedom, opportunities and health services, regardless of their sexual orientation. They adopt a universalist language that asserts the rights and privileges of all citizens and contain forthright stipulations against discrimination, criminalisation, and denial of social and economic opportunities and services to the region’s sexual minorities. A major feature of these instruments, however, is their lack of explicit mention of LGBT persons and the lack of clear mechanism by which member-states account for their implementation. Further, some states in the region have yet to assent to some of the instruments. By not specifically mentioning LGBT persons, these instruments miss an opportunity to put LGBT issues forthrightly on the front-burner of the development agenda in Africa.

However, the generalist language of these instruments, their persistent emphasis on inclusivity, and their nonmention of sexual orientation and gender identity as bases for exclusion offer critical opportunities for efforts to advance LGBT rights in the region.

Against this backdrop, the enforceable treaties are particularly key as they have provided and will continue to provide bases for court and legal pronouncements and interpretations that further elucidate the meaning and implications of regional agreements. This is amply demonstrated by the different national and regional court rulings as well as activity reports, declarations, and position statements of the different committees of the AU Commission. The available legal interpretations and statements of these instruments have affirmed sex, gender, and sexual orientation as forbidden grounds for discrimination in the region. Without exception, the instruments collectively enshrine the rights, privileges and freedoms of Africa’s LGBT persons from murder, violent attack, torture, arbitrary detention, forced marriage, denial of rights to SRH services, assembly and expression, and exclusion from education, healthcare, housing, and labour market participation. They emphasise and sanction the human rights of LGBT persons and the need to make society inclusive, safe, resilient, and sustainable for them as part of the natural diversity that characterises Africa. There is also evidence that some of these policy and legal instruments are enforceable across the region, exemplified in the use of the African Charter on Human and Peoples Rights to benchmark legal rulings in countries as wide-ranging as Botswana and Kenya. Critical national policy and programmatic work and activities in sectors such as health and education in many African countries also continue to cite the Maputo Plan of Action and the Africa Youth Charter in developing action plans, guidelines, programmes and interventions.

Going by these treaties and instruments, member-states of the AU have an inalienable obligation to protect, safeguard and ensure the inclusion of LGBT persons in the region. The instruments clearly provide a firm basis for addressing the exclusion of LGBT persons in Africa. Currently, LGBT persons are disproportionately over-represented among those who experience exclusion-related vulnerabilities in Africa. For instance, about a quarter of the homeless youth population in African cities are LGBT persons.^18,23^ Most of these youth have been forced to leave their familial homes due to conflict, including homophobia, transphobia and, most commonly, abuse and violence. When LGBT persons cannot safely access public services and spaces, they are denied the ability to enjoy healthy lives and live fully in the communities they call home. Further, violence against LGBT persons often goes uninvestigated or unpunished, which grants impunity to behaviour that endangers lives, adds to poor SRH outcomes, and makes public spaces unsafe for LGBT persons.^5,20^ The instruments are characterised by a non-discrimination principle which offer grounds for promoting LGBT access to safe, equitable housing, labour markets, health care and public services. They clearly offer a strong basis for states to adopt and pursue LGBT-specific SRH and development policies. They are, also, to a very large extent, forms of accountability frameworks against which to gauge the performance and commitment of African member-states of the AU on the protections of the inalienable rights and freedoms of its LGBT citizens to safe, healthy and inclusive lives.

## Conclusion

The SRH and other implications of the exclusion of LGBT persons are well-documented. Taken together, the instruments and policy documents discussed in this paper hold out immense promise to guide advocacy and policy engagement to ensure that continental and national development plans and policies specifically reflect the socio-economic and health needs of LGBT persons. Advocacy to address implementation gaps in these legal and policy instruments, enforce their mainstreaming in national legal and policy systems, and promote domestic accountability, is urgent. National adoption and domestication of continental policies can improve local legal environments for LGBT persons through decriminalisation, providing a sustainable pathway for inclusion and respect for LGBT persons in Africa. Effective mechanisms for peer monitoring of accountability and implementation of these instruments are also urgently needed. The AU Commission’s expert bodies and committees’ practice of making clear position statements, interpreting the instruments, and requesting regular briefings on their implementation and workings at national levels is critical and needs to be sustained. Strengthening public and civil society awareness and education regarding these instruments is another promising strategy that offers opportunities for advocacy and engagement with governments, national judicial bodies, and the African Commission to ensure that LGBT rights are continually and clearly read into these documents, either through general comments, rulings, or specific cases.
